# Enhanced Chemotherapy for Glioblastoma Multiforme Mediated by Functionalized Graphene Quantum Dots

**DOI:** 10.3390/ma13184139

**Published:** 2020-09-17

**Authors:** Giordano Perini, Valentina Palmieri, Gabriele Ciasca, Marcello D’Ascenzo, Aniello Primiano, Jacopo Gervasoni, Flavio De Maio, Marco De Spirito, Massimiliano Papi

**Affiliations:** 1 Dipartimento di Neuroscienze, Università Cattolica del Sacro Cuore, 00185 Roma, Italy; giordano.perini@unicatt.it (G.P.); gabriele.ciasca@unicatt.it (G.C.); marcello.dascenzo@unicatt.it (M.D.); marco.despirito@unicatt.it (M.D.S.); 2 Fondazione Policlinico Universitario A. Gemelli IRCSS, 00185 Roma, Italy; aniello.primiano@unicatt.it (A.P.); jacopo.gervasoni@policlinicogemelli.it (J.G.); 3 Institute for Complex Systems, National Research Council (ISC-CNR), Via dei Taurini 19, 00185 Rome, Italy; 4 Dipartimento di Scienze Biotecnologiche di Base, Cliniche Intensivologiche e Perioperatorie, Università Cattolica del Sacro Cuore, 00185 Roma, Italy; 5 Dipartimento di Scienze di Laboratorio e Infettivologiche, Fondazione Policlinico Universitario “A. Gemelli”IRCSS, 00185 Rome, Italy; flavio.demaio@unicatt.it; 6 Dipartimento di Scienze biotecnologiche di base, cliniche intensivologiche e perioperatorie—Sezione di Microbiologia, Università Cattolica del Sacro Cuore, 00185 Rome, Italy

**Keywords:** graphene, quantum dots, glioblastoma, neurons, doxorubicin

## Abstract

Glioblastoma is the most aggressive and lethal brain cancer. Current treatments involve surgical resection, radiotherapy and chemotherapy. However, the life expectancy of patients with this disease remains short and chemotherapy leads to severe adverse effects. Furthermore, the presence of the blood–brain barrier (BBB) makes it difficult for drugs to effectively reach the brain. A promising strategy lies in the use of graphene quantum dots (GQDs), which are light-responsive graphene nanoparticles that have shown the capability of crossing the BBB. Here we investigate the effect of GQDs on U87 human glioblastoma cells and primary cortical neurons. Non-functionalized GQDs (NF-GQDs) demonstrated high biocompatibility, while dimethylformamide-functionalized GQDs (DMF-GQDs) showed a toxic effect on both cell lines. The combination of GQDs and the chemotherapeutic agent doxorubicin (Dox) was tested. GQDs exerted a synergistic increase in the efficacy of chemotherapy treatment, specifically on U87 cells. The mechanism underlying this synergy was investigated, and it was found that GQDs can alter membrane permeability in a manner dependent on the surface chemistry, facilitating the uptake of Dox inside U87 cells, but not on cortical neurons. Therefore, experimental evidence indicates that GQDs could be used in a combined therapy against brain cancer, strongly increasing the efficacy of chemotherapy and, at the same time, reducing its dose requirement along with its side effects, thereby improving the life quality of patients.

## 1. Introduction

Glioblastoma multiforme (GBM) is the most aggressive and lethal human brain cancer, with poor prognosis [1]. Due to the short life expectancy of GBM patients, this tumor has drawn interest in the biomedical field and became the first one characterized by The Cancer Genome Atlas (TCGA) in 2008 [2]. Numerous reasons contribute to the ineffectiveness of current therapies against GBM. First, it is multiform, therefore it displays high intratumoral heterogeneity [3,4], which has great relevance in estimating the survival function related to diverse tumor cell subtypes [5]. Second, potentially useful drugs targeting the central nervous system (CNS) are retained in the bloodstream due to the presence of the blood–brain barrier (BBB) [6].

The BBB is the anatomical border that separates the brain from the bloodstream. This sophisticated cellular complex controls the BBB permeability of circulating molecules, including drugs [6]. Much effort in GBM research has been done in two directions, (1) by focusing on the design of molecules capable of overtaking the BBB and (2) the design of molecules that could effectively kill cancer cells [7,8,9,10,11]. A strategy gaining increasing interest lies in the use of quantum dots (QDs) [12]. QDs are semiconducting nanoparticles with excellent photophysical properties and a small size. The latter feature allows QDs to easily cross biological barriers [13]. However, most QDs lack biocompatibility due to their intrinsic cytotoxicity [14,15]. In recent years, the use of graphene quantum dots (GQDs) has spread in life sciences applications [16,17,18]. GQDs are two-dimensional materials based on graphene; they are structured as a single-atom-thick sheet of honeycomb-arranged, sp2-bonded carbon atoms and they have great electronic properties [19,20,21]. GQDs have demonstrated very good biocompatibility, having a molecule-like shape, when compared to other QDs [13]. Moreover, GQDs have shown a high capability of crossing several biological barriers [13], including the BBB, both in vitro [22,23] and in vivo [24,25]. Furthermore, experimental evidence has underlined the possible use of GQDs for cancer diagnosis and drug delivery, as well as for photothermal techniques [26,27]. In this paper, we report on the effect of GQDs functionalized with dimethylformamide (DMF-GQDs), bearing an intrinsic toxicity, and of biocompatible non-functionalized GQDs (NF-GQDs), on two neural lineages: U87 GBM cells and primary mouse cortical neurons. Previous studies pointed out the reduced tumorigenicity associated with DMF. Therefore, the functionalization of GQDs with DMF may be of interest for potential biomedical applications in cancer research [28]. Furthermore, the coadministration of GQDs and doxorubicin (Dox) is investigated. Dox is not currently employed in clinical translation due to its poor targeting ability in vivo. This could lead to adverse, toxic effects on healthy tissues. Moreover, most of the studies employing GQDs and Dox focused on the synthesis of nano-complexes by conjugating the two molecules with cancer-targeting ligands. However, the so-formed complexes were not capable of significantly reducing tumor cells viability, when compared to the drug alone [29,30]. Here we report that GQDs exert a synergistic effect along with the chemotherapeutic agent Dox. The mechanism of action behind this observation is discussed in this work by using confocal microscopy, cell viability measurements and by analyzing cellular uptake of the chemotherapeutic drug.

## 2. Materials and Methods

### 2.1. Characterization of GQDs

NF-GQDs (Sigma-Aldrich, St. Louis, MO, USA) and DMF-GQDs (ACS Materials, Pasadena, CA, USA) were optically and microscopically characterized.

Dynamic light scattering (DLS) and ζ-Potential were performed with a Zetasizer Nano ZS (Malvern, Worcestershire, UK), equipped with a 633 nm He−Ne laser, operating at an angle of 173° UV-transparent cuvettes (Malvern, Worcestershire, UK) were used for experiments with a sample volume of 600 µL and a concentration of 100 µg/mL. The measurements were performed at a fixed position (4.65 mm) with an automatic attenuator. For each sample, three measurements were averaged, the diffusion coefficient was retrieved through cumulants analysis from autocorrelation functions. The hydrodynamic radius (Z-Average size) was obtained by the Stokes−Einstein equation [31].

GQDs were deposited on sterile mica slides (TedPella, Redding, CA, USA) at a concentration of 10 µg/mL and air-dried overnight for atomic force microscopy imaging (AFM) with a NanoWizard II (JPK Instruments, Berlin, Germany). The images of small scan areas (3 × 3 µm) were obtained by using silicon cantilevers with conical silicon tips (CSC36 Mikro-Masch, Sofia, Bulgaria) characterized by an end radius of about 10 nm, a half conical angle of 20° and a spring constant of 0.6 N/m.

Fluorescence spectra were acquired by using a Cytation 3 Cell Imaging Multi-Mode Reader (Biotek, Winooski, VT, USA) exciting from 260 to 600 nm and reading the emission from 300 to 700 nm.

Surface chemical analysis of GQDs was performed using attenuated total reflectance-Fourier transform infrared spectroscopy (ATR-FTIR) with a Spectrum One spectrometer (Perkin Elmer, Waltham, MS, USA). The material under investigation was laid upon the ATR crystal and the spectra were recorded in the wave number range of 4.000 to 550 cm^−1^.

### 2.2. Cell Culture

U87 human GBM cells were purchased from the American Type Culture Collection, (ATTC, Manassas, VA, USA). Cells were maintained in Dulbecco’s modified Eagle’s medium (DMEM) (Sigma-Aldrich, St. Louis, MO, USA), supplemented with 10% foetal bovine serum (FBS, EuroClone, Milan, Italy), 2% penicillin-streptomycin (Sigma-Aldrich, St. Louis, USA) and 2% L-glutamine (Sigma-Aldrich, St. Louis, MO, USA).

Primary cultures of cortical neurons were obtained from E15-18 C57BL/6 mice embryos as described previously and in accordance with the Ethics Committee of the Catholic University and in compliance with the Italian Ministry of Health guidelines, with national laws (Legislative Decree 116/ 1992) and European Union guidelines on animal research (No. 86/609/EEC) [32]. Briefly, the mouse cortex was dissected in cold CMF-HBSS (Ca^2+^ and Mg^2+^ free Hank’s balanced salt solution containing 1 mM pyruvate, 15 mM HEPES, and 10 mM NaHCO_3_, Sigma-Aldrich, St. Louis, MO, USA). Tissues were then incubated for 10 min at 37 °C in PBS containing trypsin-ethylenediaminetetraacetic acid (0.025%/0.01% wt/vol; Biochrom AG, Cambridge, UK), and the tissue was mechanically dissociated at room temperature with a fire-polished Pasteur pipette. The cell suspension was harvested and centrifuged at 235 g for 8 min. The pellet was suspended in 88.8% Minimum Essential Medium (Biochrom, Cambridge, UK), 5% FBS, 5% horse serum, 1% glutamine (2 mM), 1% penicillin-streptomycin-neomycin antibiotic mixture (Invitrogen, city, country), and glucose (25 mM). Cells were plated at a density of 10^5^ cells/mL on a 24-well plate precoated with poly-L-lysine (0.1 mg/mL; Sigma-Aldrich, St. Louis, MO, USA). Twenty-four hours later, the culture medium was replaced with a mixture of 96.5% Neurobasal medium (Invitrogen, Carlsbad, CA, USA), 2% B-27 (Invitrogen, Carlsbad, CA, USA), 0.5% glutamine (2 mM), and a 1% penicillin-streptomycin-neomycin antibiotic mixture. After 72 h, this medium was replaced with a glutamine-free version of the same medium, and the cells were grown for 10 more days before experiments were conducted. All cell lines were cultivated in T75 flasks and kept at 37 °C in 5% CO_2_.

### 2.3. Cell Viability Measurements

Human GBM cells and primary cortical neurons were cultured on 96-well flat bottom plates (Corning, New York, NY, USA), at a concentration of 1x10^4^ cells/well, with a volume of 100 µL. Cells were incubated at 37 °C. Twenty-four h after seeding, cells were treated with GQDs at four concentrations: 250, 200, 100 and 50 µg/mL, and incubated. After 24 h, the medium was replaced with a fresh culture medium (100 µL), and with an addition of 10 µL of 3-(4,5-dimethylthiazol-2-yl)-2,5-diphenyltetrazolium bromide (MTT, Invitrogen, Carlsbad, CA, USA) at a concentration of 12 mM. After 4 h of incubation, 100 µL of sodium dodecyl sulfate (SDS, Invitrogen, Carlsbad, CA, USA), dissolved in 0.01 M HCl (Sigma-Aldrich, St. Louis, MO, USA), was added to each well, and incubated at 37 °C in 5% CO_2_ for 16 h. Absorbance was read at 570 nm, and data were normalized by control (untreated) cells.

For treatment, after incubation with GQDs, the medium was removed, washed with PBS and replaced with a fresh culture medium containing Dox 1 µM. After 48 h of incubation, MTT was performed as reported.

### 2.4. Confocal Microscopy

To perform confocal microscope analysis, GBM cells and cortical neurons were plated on sterile chamber slides (Ibidi, Gräfelfing, Germany) at a concentration of 1 × 10^6^ cell per mL in a final volume of 300 μL and then incubated at 37 °C. After 24 h, GQDs were administered to cells at the concentration of 250 μg/mL for a further 24 h. For both 6-dodecanoyl-2-dimethylamino-naphthalene (Laurdan) measurements and Dox uptake (Sigma-Aldrich, St. Louis, MO, USA), GQDs were carefully washed away with PBS, and the cells were resuspended in a fresh medium containing laurdan or Dox. For laurdan measurements, a stock solution of laurdan 1 mM in dimethyl sulfoxide (DMSO) was diluted 1:1000 in DMEM and administered to the cells. For Dox uptake measurements, the Dox was diluted from a stock solution at a concentration of 1.5 mM to a final concentration of 1 μM in DMEM, and administered to cells.

Confocal microscopy measurements of laurdan and Dox uptake were carried out using an inverted microscope (Nikon A1 MP+, Nikon, Tokyo, Japan) equipped with a 60× oil immersion objective. Images were acquired at 37 °C. Laurdan intensity images were recorded with emissions in the range of 425 to 475 nm (gel-phase) and 500 to 550 nm (liquid-phase). 

For Dox uptake measurements, images were acquired after 1 h of incubation with Dox. The excitation and emission wavelengths were 488 nm and 590 nm, respectively. To quantify both laurdan fluorescence and Dox uptake, the Fiji (ImageJ) software (Bethesda, MD, USA) was used [33].

## 3. Results and Discussion

**Figure 1:**
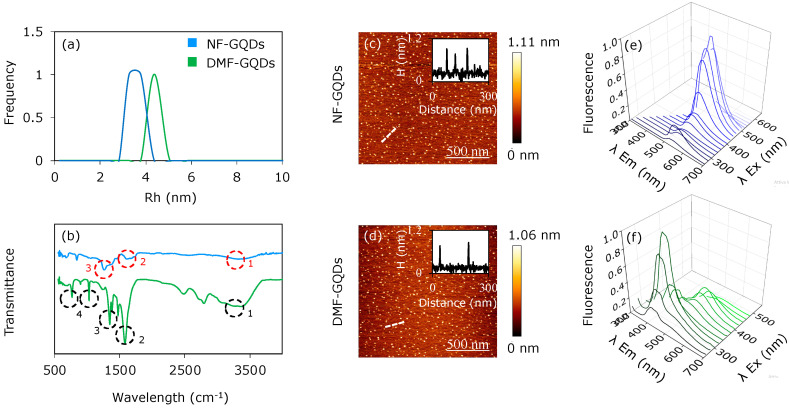
Characterization of graphene quantum dots (GQDs). Dynamic light scattering (DLS) of non-functionalized GQDs (NF-GQDs) and dimethylformamide-functionalized GQDs (DMF-GQDs (**a**). Fourier transform infrared spectroscopy (FTIR) spectra of NF-GQDs and DMF-GQDs (**b**). Atomic force microscopy imaging (AFM) imaging of NF-GQDs (**c**) and DMF-GQDs (**d**), with relative line profile (insets). Dashed lines show the image regions corresponding to the line profiles in the insets. Optical characterization by fluorescence emission spectra of NF-GQDs (**e**) and DMF-GQDs (**f**).

Figure 1 depicts optical and microscopic characterization of DMF-GQDs and NF-GQDs. DLS measurements (Figure 1a) show a hydrodynamic radius below 10 nm, in accordance with literature for QDs [16], by taking into account the geometry of two-dimensional graphene-based materials [34]. ATR-FTIR spectra were acquired for both nanoparticles (Figure 1b). The infrared (IR) spectra of NF-GQDs show a band at 3333 cm^−1^ due to O–H stretching vibrations, a band at 1613 cm^−1^ due to the C=C bond [35] and a 1260 cm^−1^ band corresponding to epoxyde C–O stretching vibrations (Figure 1b, indicated with red circles numbered 1, 2 and 3, respectively) [36]. The IR spectra of DMF-GQDs show a broadening of the vibration modes at 1575 cm^−1^, related to the aromatic structure [35]. The band at 1467 cm^−1^ is due to stretching and deformation vibrations of the C–H in methylene groups (Figure 1b, black circles numbered 2 and 3) [37]. The minor signal at 1227 cm^−1^ could be due to C-OH bond and the signal at 1024 cm^−1^ is caused by oxidative carbon species (epoxides, carboxyls, etc., indicated by black circle number 4 in Figure 1b) [36].

AFM imaging was performed on each sample (Figure 1c,d) and representative cross sections (Figure 1c,d insets) are reported, showing a lateral size below 10 nm [34]. 

Fluorescence emission spectra of both nanoparticles were acquired in a range of excitation wavelengths from 360 to 500 nm (Figure 1e,f), with a step of 20 nm. NF-GQDs show an emission peak at 530 nm when excited at 460 nm. DMF-GQDs show two main emission peaks: the first at 420 nm, by exciting at 320 nm; the second, less intense, at 530 nm, by exciting at 420 nm. The second peak is in accordance with previously reported DMF-GQDs [38]. Interestingly, the blue peak at 420 nm for DMF-GQDs could be due to the presence of N and O atoms on the DMF groups [39]. It is known that O atoms lead to the formation of small sp^2^ clusters along graphene sheets, which are responsible for blue emission [40]. Furthermore, previous experimental and theoretical observations have proven the strong electron-withdrawing ability of the N atoms: therefore, the strong electron affinity of the N atoms could contribute to the blue emission as well [39].

**Figure 2:**
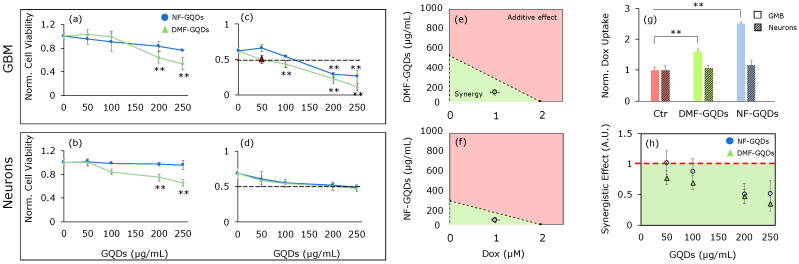
Biocompatibility and effect of GQDs in enhanced chemotherapy. Cell viability of GQDs on glioblastoma multiforme (GBM) (**a**) and cortical neurons (**b**) normalized by untreated cells. Viability after GQDs enhanced chemotherapy on GBM (**c**) and cortical neurons (**d**). Isoboles of enhanced chemotherapy on GBM for DMF-GQDs (**e**) and NF-GQDs (**f**). Uptake of doxorubicin (Dox) in untreated GBM cells, and after the treatment with DMF and NF-GQDs (**g**). The synergy of Dox and GQDs at the tested concentrations on GBM and neurons (**h**). The data are expressed as the mean values normalized to the untreated cells ± standard deviation. Synergy is expressed as the ratio between the theoretical additive effect of the two molecules and the combined measured effect. ** *p* < 0.01, one-way ANOVA and Turkey post-hoc test.

Figure 2a,b displays the 24 h cell viability results of U87 GBM cells and cortical neurons normalized by control (untreated) cells after the administration of GQDs at different concentrations (50, 100, 200 and 250 µg/mL). NF-GQDs demonstrated good biocompatibility on both cell lines, showing a mild reduction of GBM cell viability at 250 µg/mL. DMF-GQDs reduce cell viability, especially of GMB cells, to 0.53 ± 0.09 at 250 µg/mL. We then tested the effect of the combined therapy with GQDs and Dox. Initially, the concentration of drug inhibiting 50% of cell growth (IC_50_) of Dox was measured on GBM cells; the result was 2 µM (data not shown) [41,42].

For combined therapy, cells were treated with GQDs for 24 h. We then washed the cells with PBS to remove nanoparticles which had not interacted with the cells. We next replaced the medium with fresh medium containing Dox at 1 µM (half of its IC_50_). Figure 2c,d shows cell viability normalized to the control (untreated) cells after administration of GQDs and Dox. 

The combined treatment with Dox significantly decreased the GBM viability only at high concentrations of NF-GQDs, with viability at 0.30 ± 0.08 at 200 µg/mL and 0.27 ± 0.09 at 250 µg/mL, respectively (Figure 2c). DMF-GQDs combined with Dox did not affect the viability of cortical neurons significantly, however the combined treatment caused a strong reduction of cell numbers at 250 µg/mL. Interestingly, DMF-GQDs exerted a strong toxic effect with Dox on GBM cells even at low concentrations (Figure 2c, 50 and 100 µg/mL). In particular, DMF-GQDs at 50 µg/mL combined with Dox, reduced GBM viability as would the IC_50_ of the chemotherapeutic drug alone. Furthermore, a significant decrease in cell viability was measured as 0.43 ± 0.06 by combining Dox and DMF-GQDs at 100 µg/mL. At 50 and 100 µg/mL, DMF-GQDs showed good biocompatibility on cortical neurons and there was no evident toxicity. Therefore, a very low concentration of DMF-GQDs, combined with Dox at half of its IC_50_, could strongly increase the efficacy of the latter synergistically, reducing doses needed for chemotherapy. NF-GQDs combined with Dox did not significantly reduce the viability of cortical neurons when compared to the treatment with Dox alone (Figure 2d). 

Hence, the synergistic enhanced chemotherapy of NF-GQDs and DMF-GQDs was investigated. Figure 2e,f displays isoboles of NF-GQDs and DMF-GQDs along with Dox, respectively, on GBM. In isoboles, the IC_50_ of GQDs and Dox are reported separately on the axes and are connected by a line. This line defines two areas in which there is additivity (above the line) or synergism (below the line). The IC_50_ of the combined treatment is plotted as a single point. The combined treatment can be considered synergistic or additive, depending on where the point falls [43]. Both of the GQDs, when combined with Dox, exerted a synergistic effect. 

**Figure 3:**
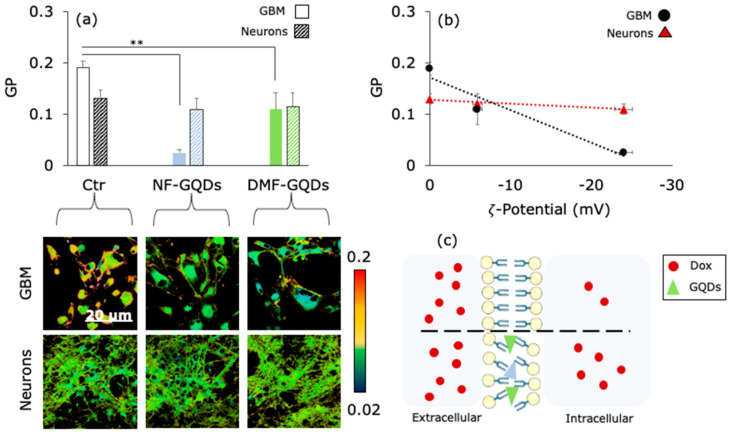
Synergistic mechanism of GQDs and chemotherapy. GP values of GBM and neurons, obtained with Laurdan confocal imaging, show an increase in membrane permeability after the treatment with GQDs (**a**). In the inset, representative images obtained with confocal microscopy. The correlation between the surface net charge of GQDs and GP on GBM (**b**). The proposed mechanism for GQDs enhanced chemotherapy (**c**). ** *p* < 0.01, one-way ANOVA and Turkey post-hoc test.

To explain the GQDs’ enhanced chemotherapy effects, the uptake of Dox in cells was tested by confocal microscopy (Figure 3) [29]. An increased uptake of Dox after the administration of GQDs on GBM cells was observed. This result indicates that enhanced chemotherapy was due to an increased uptake of the antitumor drug specifically inside GBM cells. Figure 2h shows the ratio between the combined measured effect of GQDs and Dox and the theoretical additive effect on GBM. The lower the ratio, the higher the synergy between the two molecules. A ratio equal to 1 indicates an additive effect, while higher values could point out antagonistic effects. Only high concentrations of NF-GQDs (200 and 250 µg/mL) combined with Dox result in a synergistic effect. DMF-GQDs at all concentrations exert a synergistic effect, further highlighting its potential application at low biocompatible concentrations in a combined therapy with a reduced dose of antitumor drugs.

Previous studies hypothesized that QDs could increase cellular uptake of antitumor drugs by increasing membrane permeability of cancer cells [29]. However, direct measurements of changes in membrane permeability have not been reported so far. To measure changes in membrane permeability, cells were labeled with laurdan, a polar sensitive fluorescent membrane probe [44]. Laurdan has a dipole moment so, when excited, it can rearrange the surrounding solvent in an energy dispersant process (dipolar relaxation) that causes red shift in its emission [45]. Therefore, laurdan can distinguish between the gel and liquid phases of solvent. To quantify membrane permeability, the generalized polarization (GP) measure has been used, expressed as the ratio between the emission range of laurdan in a gel phase (425–475 nm) and in a liquid phase (500–550 nm); the lower the GP, the higher the cell fluidity [45]. Figure 3a shows a significant increase in membrane fluidity for GBM treated with GQDs when compared to the control (untreated) cells, while changes in cortical neurons GP were not measured. Representative laurdan intensity images are reported in the insets (Figure 3a, bottom). The interaction between the cell membrane and the GQDs depends on the size and surface chemistry of the nanoparticles [24,46]. Previous studies highlighted that cationic QDs did not enter inside cells, indicating that QDs with a more cationic surface tend to interact with paracellular pathways, while anionic nanoparticles are known to be incorporated into cells mainly by endocytosis [47,48]. By measuring the zeta potential of the two GQDs, a correlation between cell-specific change in membrane permeability and the surface net charge of the GQDs (Figure 3b) was observed. Previous studies tested the combination of Dox and GQDs. Complexes made of the two molecules were used and functionalized with several specific ligands of receptors overexpressed in tumors. Wang et al. synthesized GQDs functionalized with folic acid and conjugated with Dox [49]. They investigated the specific delivery of chemotherapy to HeLa cells, in which the receptor of folic acid in overexpressed, with respect to other cell lines (e.g., HEK293A, A549). Reduction in cell viability was measured for HeLa cells, but not on the other cell lines. However, the formation of complexes could be unstable, and could reduce the efficacy of the chemotherapeutic agent itself. Sui et al. measured the uptake of cisplatin in different cell lines in the coadministration with GQDs [29]. They found an increased killing effect for cisplatin coadministered with GQDs when compared to a free drug. Another kind of interaction that could contribute to change membrane permeability resides in the Van der Waals attraction between the GQDs and the lipid bilayer. Theoretical investigations showed the capability of graphene to interact with biological membranes. Molecular dynamics simulations on graphene and the Escherichia Coli membrane pointed out the Van der Waals attractions between lipid heads and the edge of the graphene, which was strongly dependent on the surface net charge of the graphene edges [50]. After a few nanoseconds, insertion of graphene between the membrane, like a sandwich, occurred, along with a correspondent change in permeability. The same insertion happened by simulating micelles and graphene nanosheets [51]. Although an increase in the efficacy of chemotherapy has been observed, direct measurements of the mechanism of action involving membrane permeability have not been reported so far. The mechanism of action of GQDs that emerges from our experimental evidence is reported in Figure 3c. GQDs interact with the cell membrane of GBM, changing the membrane fluidity. The surface chemistry of GQDs strongly influences this interaction, promoting the entrance of negatively charged molecules [22]. GQDs tend to interact with the cell membrane in a cell-specific, surface chemistry-dependent manner [47]. This interaction alters the membrane permeability of the GBM cells, but not of the cortical neurons, specifically increasing the uptake and efficacy of Dox.

## 4. Conclusions

GQDs are showing their potential in the biomedical field, becoming excellent candidates for several applications especially to the brain, thanks to their ability to cross the BBB.

Here it was found that NF-GQDs were biocompatible at all the tested concentrations. DMF-GQDs, on the contrary, were toxic at high concentrations, in particular for GBM cells. However, at lower concentrations (100 and 50 µg/mL), DMF-GQDs resulted in being biocompatible on both cell lines. When combined with Dox, GQDs significantly affected the cell viability of the GBM cells. DMF-GQDs, at all the concentrations, exerted a synergistic effect with the antitumor drug, increasing its effectiveness. In particular, the combination with DMF-GQDs at 50 µg/mL reduced cell viability to the corresponding IC_50_ of Dox, and the combination with DMF-GQDs at the still biocompatible concentration of 100 µg/mL significantly affected viability at levels lower than the IC_50_ of the antitumor drug alone. The underlying mechanism was mediated by an increase in the uptake of Dox due to cell-specific changes in the membrane permeability of U87 cells. Changes in the membrane permeability were correlated to the surface charge of the GQDs. Taken together, these results indicate that biocompatible NF-GQDs could be used in a combined therapy with chemotherapeutics at lower concentrations. Importantly, this piece of evidence indicates that DMF-GQDs, at biocompatible concentrations, could be excellent candidates in a combined therapy, strongly reducing the dose of the antitumor drug.
